# How many women take oral supplementation in pregnancy in Austria? Who recommended it? A cross-sectional study

**DOI:** 10.1007/s00508-019-1502-9

**Published:** 2019-05-16

**Authors:** Ulrike Spary-Kainz, Thomas Semlitsch, Sophie Rundel, Alexander Avian, Sereina Herzog, Heidelinde Jakse, Andrea Siebenhofer

**Affiliations:** 1 Institute of General Practice and Evidence-based Health Services Research, Medical University of Graz, Auenbruggerplatz 20/III, 8036 Graz, Austria; 2 Institute for Medical Informatics, Statistics and Documentation, Medical University of Graz, Graz, Austria; 3 Steiermärkische Gebietskrankenkasse (Styrian State Health Insurance Fund), Graz, Austria; 4 Institute of General Practice, Goethe-University Frankfurt am Main, Frankfurt am Main, Germany

**Keywords:** Anemia, Iron deficiency, Pregnancy, Nutrition therapy, Preventive health services

## Abstract

**Background:**

Iron deficiency anemia is common in pregnancy with a prevalence of approximately 16% in Austria; however, international guideline recommendations on screening and subsequent treatment with iron preparations are inconsistent. The aim of this study was to find out how often pregnant women take iron-containing supplements, and who recommended them. As hemoglobin data were available for a sub-group of women, hemoglobin status during pregnancy and associated consumption of iron-containing medications were also recorded.

**Methods:**

This cross-sectional study was conducted at the Mother-Child-Booklet service center of the Styrian Health Insurance Fund in Graz, Austria. A questionnaire containing seven questions was developed. Absolute and relative numbers were determined, and corresponding 95% confidence intervals calculated using bootstrapping techniques.

**Results:**

A total of 325 women completed the questionnaire, 11% had been diagnosed with anemia before becoming pregnant, 67% reported taking iron-containing compounds. The women reported taking 45 different products but 61% took 1 of 3 different supplements. Overall, 185 (57%) women had not been diagnosed with anemia before becoming pregnant but reported taking an iron-containing supplement and 89% of the women took supplements on the recommendation of their physician. Of the 202 women whose hemoglobin status was assessed, 92% were found not to be anemic.

**Conclusion:**

Overall, 67% of pregnant women took iron-containing compounds, irrespective of whether they were deficient in iron. Physicians were generally responsible for advising them to take them. No standardized procedure is available on which to base the decision whether to take iron during pregnancy, even in guidelines. As most guidelines only recommend taking iron supplements in cases of anemia, the high percentage of women taking them in Austria is incomprehensible.

**Electronic supplementary material:**

The online version of this article (10.1007/s00508-019-1502-9) contains supplementary material, which is available to authorized users.

## Introduction

Iron deficiency anemia is the most common form of anemia in pregnancy and is usually hypochromic and microcytic [1] and associated with a low level of ferritin. According to the World Health Organization (WHO), its prevalence during pregnancy is approximately 15.5% in Austria [2]. During a singleton gestation, the blood volume expands by approximately 50% and total red blood cell mass by around 25%. The need for iron increases accordingly [3]. The definition of anemia varies during the course of pregnancy and is defined as a hemoglobin level < 11.0 mg/dl in the first trimester, 10.5 mg/dl in the second trimester and again 11 mg/dl in the third trimester [2].

Several international guidelines recommend regular anemia screening [4–6], while two guidelines only recommend screening in the case of an existing indication [7, 8]. Due to a paucity of evidence, one guideline [9] does not recommend anemia screening at all. There are also huge differences in recommendations whether to take iron supplements.

During pregnancy, the American Congress of Obstetricians and Gynecology (ACOG) recommends taking 27 mg of dietary ferrous iron daily [3], whereas the UK guidelines do not routinely recommend iron supplementation. In the UK, an individualized approach is taken based on the results of blood count screening and the identification of women at increased risk due to previously confirmed anemia, multiple pregnancies, pregnancies within 1 year of one another and vegetarianism [10]. Guidelines from other developed countries do not recommend routine iron supplementation at all [2, 4, 6, 7]. In contrast, the WHO recommends that all pregnant women take 30–60 mg iron daily. For women living in a region where anemia is a severe public health problem, the daily intake of 60 mg of elemental iron is recommended, beginning as early as possible [11]. Iron supplementation leads to a fall in the prevalence of maternal anemia at delivery [12]; however, it is unclear if iron supplementation in well-nourished non-anemic pregnant women affects perinatal outcomes [3].

This study is part of a project dealing with the usefulness of repeated anemia screening during pregnancy. Since 1974, the Austrian government has required certain test results for mothers and their newborns to be entered into a so-called Mutter-Kind-Pass (mother-child booklet; MCB) until the child is 62 months old [13]. As a result of new developments in research, this program is now under review by the Austrian Ministry of Health [14]. During a structured assessment of existing guidelines [15] and discussions by a panel of experts, the question arose whether the two blood samples (before week 16 and in weeks 25–28) that are currently required during pregnancy are actually necessary. Currently, these screening tests check blood hemoglobin and the number of erythrocytes but not the level of ferritin [13]. A project consisting of two work packages has therefore been initiated. To determine the prevalence of anemia during pregnancy, the first package involves retrospective cohort analyses of hemoglobin levels measured in week ≤ 16 and between weeks 25–28 in pregnant women who had both tests at the MCB service center of the Styrian Health Insurance Fund (Steiermärkische Gebietskrankenkasse). This package is currently ongoing. The second package, which is described in this article, investigated how often and why women took iron and iron-containing multivitamin supplements during pregnancy. The results of the whole project will be reported to the panel of experts to enable them to develop appropriate recommendations for a new MCB program to be implemented by 2019.

In addition to how often pregnant women take iron and iron-containing multivitamin supplements during pregnancy, the aim of this study was to determine when they began taking them and on whose recommendation. As hemoglobin data were available for a limited group of women, the hemoglobin status during pregnancy and associated consumption of iron-containing medications were also recorded.

## Patients, material and methods

### Patients and procedures

This cross-sectional study was conducted at the MCB service center of the Styrian Health Insurance Fund in Graz, Austria, where women come to perform oral glucose tolerance tests and provide a second blood sample for anemia screening and other tests. To fulfill inclusion criteria, participants had to be at least 18 years old and speak German or English. The women were asked to fill in an anonymous questionnaire and, with their consent, provided data on hemoglobin values (from both the 1st and 2nd laboratory tests).

A questionnaire containing seven questions was developed by the study team, a psychologist, and an expert from the MCB service center. The MCB service center then pilot-tested the questionnaire for comprehensibility. The women were asked to provide some personal details and to confirm whether they were already taking a multivitamin supplement or had already been diagnosed with anemia. In addition, women already taking an iron or multivitamin supplement were asked to provide information on the brand name of the drug, how often they took it and when they had commenced treatment. For further details, see full questionnaire in Supplemental Fig. 1 (questionnaire for pregnant women). All patients were asked to provide written consent to participate in the study. From October 2016 to February 2017, a medical student under the supervision of a physician from the MCB service center attended the outpatient clinic once a week and asked women to provide consent and to fill in the questionnaire.

**Fig. 1:**
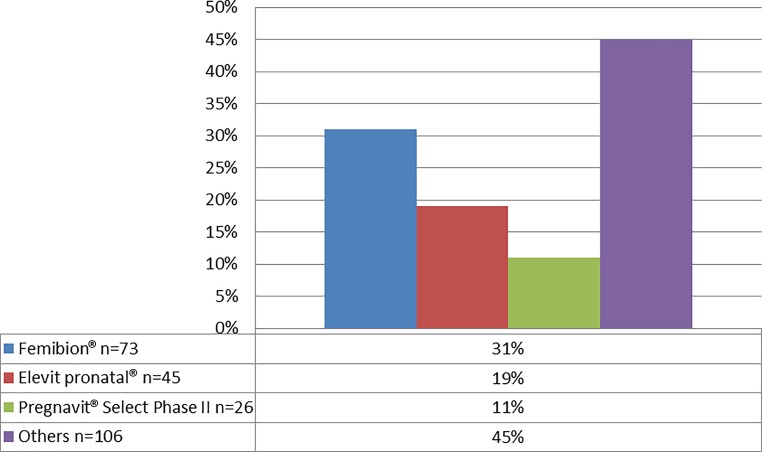
Percentage of women taking specific medications, multiple answers possible (*n* = 237)

### Sample size calculation

The sample size calculation was based on a pre-examination of 70 pregnant women of whom 13% (*n* = 9) had been diagnosed with anemia before becoming pregnant and 75% (*n* = 46) of the remaining 61 women were already taking an iron or multivitamin supplement. With a sample size of 289, a 2-sided 95% confidence interval for a single proportion will extend 0.05 from the observed proportion, for an expected proportion of 0.75. Assuming a drop-out rate of 10%, 322 pregnant women were included to reach the minimum targeted sample size of 289.

### Statistical analysis

The absolute and relative number of women who reported taking iron-containing medication was determined. Corresponding 95% confidence intervals were calculated using bootstrapping techniques. This analysis was performed for (1) all women and for (2) women without confirmed anemia before becoming pregnant. The sub-group of women for whom data on hemoglobin values were reported in their MCB booklets (1st examination: week ≤ 16 and 2nd examination: between weeks 25–28) were analyzed using absolute and relative numbers. Anemia was defined as a hemoglobin level (Hb) < 11 mg/dl in accordance with the WHO definition [16].

## Results

Overall, 325 women completed the questionnaire. Of these 62.1% were over 30 years of age and it was the first pregnancy for 51.7%. In all, 10.8% (*n* = 35) of the women had been diagnosed with anemia before becoming pregnant, 33 were taking medication, of whom 6 were taking iron intravenously and 5 had changed their diet. Of all the analyzed women, 72.9% (237/325, 95% CI [Confidence Interval]: 67.7–77.8%) reported taking an iron, a multivitamin plus iron, or a multivitamin supplement during pregnancy (Table 1). Overall, the women were taking 45 different products, of which 61% were either Femibion® 1 and 2 (Merck, Darmstadt, Germany), Elevit® pronatal (Bayer, Leverkusen, Germany) or Pregnavit® Select Phase II (Ratiopharm, Ulm, Germany) (Fig. 1). Femibion® 1 and 2 contain 28 mg of iron, whereas Elevit® prenatal contains 60 mg and Pregnavit® Selecht Phase II 15 mg (Table 2). Of the women 6 did not report which product they took and a further 12 took a medication that contained no iron. Thus, 67.4% (219; 95%CI: 62.5–72.3%) of all women and 64.5% (185/287; 95%CI: 58.8–69.8%) of women who had not been diagnosed with anemia before becoming pregnant reported taking an iron-containing supplement.

**Table 1:** Characteristics of the pregnant women

Characteristics (*n* = 325)	*n* (%)
**Age**	
<20 years	4 (1.2)
20–29 years	119 (36.6)
30–35 years	134 (41.2)
>35 years	68 (20.9)
**Number of pregnancies**	
First	168 (51.7)
Second	103 (31.7)
Third	34 (10.5)
Fourth	11 (3.4)
>Fourth	8 (2.5)
Missing	1 (0.3)
**Anemia before pregnancy**	
Yes	35 (10.8)
No	287 (88.3)
Missing	3 (0.9)
**Supplementation**	
Iron	222 (68.3%)
Iron plus multivitamin	10 (3.1%)
Multivitamin only	5 (1.5%)
No	85 (26.2%)
Missing	3 (0.9%)

**Table 2:** Iron content according to package leaflet

Brandname	Iron content	Vit. C
Pregnavit® Select Phase II (Ratiopharm, Ulm, Germany)	15 mg	+
Femibion® 1 (Merck, Darmstadt, Germany)	28 mg	+
Femibion® 2 (Merck, Darmstadt, Germany)	28 mg	+
Elevit® pronatal (Bayer, Lerverkusen, Germany)	60 mg	+
Ferretab® (Gerot-Lannach, Lannach, Austria)	100 mg	+

Iron content according to package leaflet 14 September 2017

Of the 185 women who had not been diagnosed with anemia before becoming pregnant, 78.4% were taking an iron-containing supplement regularly, 19.5% irregularly and 2.2% did not answer this question. In total, 88.6% (164/185) took supplements because a physician recommended doing so, 11.9% (22/185) on the recommendation of family or friends, and 4.9% (9/185) on the advice of a pharmacist. In addition, 46.5% (86/185) started taking them before their first MCB hemoglobin test and 28.1% (52/185) before they knew they were pregnant. Only 24.9% (46/185) started taking an iron supplement following the analysis of the first blood sample (Table 3).

**Table 3:** Characteristics of women without confirmed anemia before becoming pregnant who take iron supplements

Characteristics (*n* = 185)	*n* (%)
Recommendation to take supplement^a^	
Medical doctor	164 (88.6)
Family and friends	22 (11.9)
Pharmacist	9 (4.9)
Others	6 (3.2)
Time women started taking supplement	
Before pregnancy	52 (28.1)
Before first blood sample	86 (46.5)
After first blood sample	46 (24.9)

^a^Multiple answers possible

**Fig. 2:**
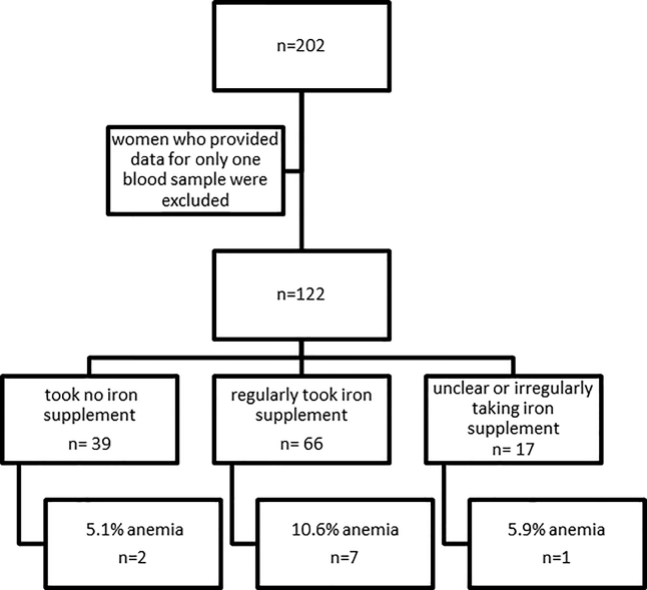
Flow chart of women providing hemoglobin level test results during pregnancy

**Fig. 3:**
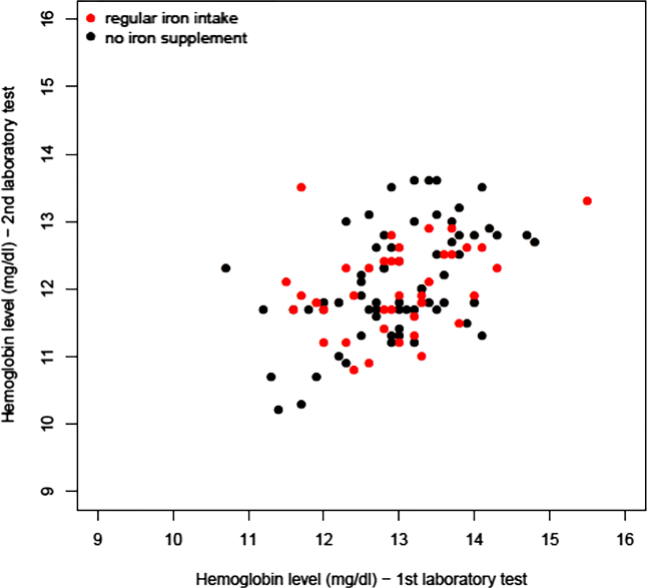
Scatterplot of second compared to first hemoglobin levels obtained before gestational week 16 and between weeks 25 and 28 in the 122 women with information on both, stratified by iron supplementation

In the second part of the study involving 202 women (Fig. 2), hemoglobin data from the first and second blood measurements were available for 122 women. Of these, 112 (91.8%) had no anemia on either occasion. In 8 women (6.6%), anemia was only detected the second time, while 1 (0.8%) woman was diagnosed as anemic only after the first blood test, and another following both (Supplemental Table 1). The lowest detected hemoglobin level in the first blood test was 9.5 mg. This woman was already taking an iron supplement (Ferretab®, Gerot Lannach, Lannach, Austria) and also had the lowest hemoglobin level in the second blood test (8.3 mg). At this time, she was still taking one of the iron supplements mentioned above. Figure 2 shows that 10.6% (*n* = 7/66) of women regularly taking iron supplements had anemia, compared to 5.1% (*n* = 2/39) of women taking no iron product. Since the number of women with anemia was very low tests for significance were not performed. This result should therefore be regarded as descriptive. Figure 3 shows separately the change in hemoglobin values for both women taking iron supplements, and women not taking iron supplements. The scatterplot reveals that there were comparable changes in both groups.

## Discussion

This cross-sectional study showed that of 325 pregnant women surveyed at the MCB service center of the Styrian Health Insurance Fund in Graz, only 11% had been diagnosed with anemia before becoming pregnant; however, an iron supplement was taken by 67% of women. Of the women that had not been diagnosed with anemia before becoming pregnant, 89% were advised to do so by their physicians. For the limited group of women for whom hemoglobin data were available for both examinations (*n* = 122), over 90% had no anemia before pregnancy, and in approximately 7%, anemia was only diagnosed in the second blood test (weeks 25–28). In one woman, anemia was only diagnosed in the first test (week ≤ 16), and in one the result was positive in both tests.

To the best of our knowledge, no study in Austria has yet asked pregnant women whether they take iron or iron-containing multivitamin supplements. As far as the questionnaire is concerned, the predefined study sample size was reached, and the level of missing data was low. In addition, women at the MCB service center were asked to fill in the questionaire, and the number of women who refused this was exceptionally low. It must be acknowledged that the analysis of the different hemoglobin values in the first and second trimester should be regarded as descriptive.

Guideline recommendations in different countries diverge substantially from one another. A look at high-income countries also shows considerable differences, with e.g. UK guidelines on the management of iron deficiency in pregnancy recommending no routine iron supplement for pregnant women [10]. The Austrian Society for Gynecology and Obstetrics also recommends only taking supplements when iron deficiency or iron deficiency anemia has been proven [2]. In contrast, the WHO, which also takes low-income countries into consideration, recommends that pregnant women routinely take iron supplements [11]. The recommended dose of iron ranges from 30 to 60 mg. In areas where anemia is a severe public health problem (defined as a prevalence of 40% or more), a daily dose of 60 mg of iron is preferred [17]. As a result, the WHO recommends that women in Austria, where the prevalence of iron deficiency anemia in women is 15.5%, take 30 mg iron [2]. There are good reasons to recommend that pregnant women from low and middle income countries, where multiple micronutrient deficiencies exist, take supplements containing iron and folic acid, as well as multiple micronutrients [17]. Although supplements reduce the risk of maternal anemia and iron deficiency in pregnancy, positive effects on other maternal and infant outcomes are less clear.

Observance of iron supplementation recommendations produces heterogeneous results, depending on the risk of low birth weight and anemia in the country concerned, as well as the level of adherence [12]. Gastrointestinal side effects are the most common complaint among women consuming high doses of iron and include constipation, nausea, vomiting, and diarrhea [17]. A further interesting finding was that over 61% of the women under review took 3 different supplements, all of which contained different levels of iron (Fig. 1 and Table 3). Whereas two of these contained a dose of around or below the recommended prophylactic regimen, the third contained therapeutic doses of iron. It appears that physicians’ and pharmacists’ recommendations are not evidence-based but made arbitrarily and possibly influenced by corporate marketing strategies. In Austria, women with no confirmed diagnosis of iron deficiency have to pay for iron and multivitamin medications themselves. The cost of taking the three most popular products from week five until childbirth is approximately €167 for Femibion® (Merck), €164 for for Elevit® pronatal (Bayer) and €140 for Pregnavit® Select Phase II (Ratiopharm) (Supplemental Table 2). Some of the women interviewed at the MCB service center said they took Elevit® pronatal (Bayer) because a private insurance company reimburses the cost of this brand. It is unclear why this insurance company pays only for this preparation and not for others containing less iron, as recommended by the WHO for high-income countries [11].

In this study, it was found that 91.8% of the women who provided information on hemoglobin levels (112/122) were not anemic. Anemia was found to be present in 5.1% (2/39) of those taking no iron supplements and 10.6% (7/66) of those taking them. This result indicates that the decision whether to take iron supplements is made haphazardly. This is also reflected in the scatter plot (Fig. 3); however, especially in a country such as Austria, where the prevalence of anemia is low and laboratory analyses are easily available, iron supplementation should perhaps be based on laboratory results. As the screening of iron/ferritin levels is the gold standard for the diagnosis of anemia, it would also make sense to go one step further and to use this test to detect and monitor iron deficiency anemia; however, to the best of our knowledge, the new MCB program plans only the continued measurement of hemoglobin values.

There are several limitations to this study. Firstly, the questionnaire was only piloted in a small group of women at the MCB center and no extensive validation was performed. In addition, only a limited group of women were questioned at one service center in Graz, However, the center is run by a statutory health insurer and is attended by women of all social categories. It is uncertain to what extent these results are representative of the whole of Austria.

A high percentage of a limited study group of pregnant Austrian women were routinely taking iron-containing compounds, irrespective of whether they had iron deficiency anemia. In general, they had been advised to take the supplements by their physicians. In high-income countries such as Austria, most guidelines only recommend taking iron supplements in cases of anemia. The high percentage of women taking them and the lack of any standardized procedure on which to base the decision whether to take them is therefore incomprehensible. Based on these results and those of the ongoing retrospective analysis on the prevalence of anemia in pregnant women in Austria, it should be possible to advise the panel of experts of the Austrian Health Ministry whether one or two blood tests for anemia are necessary. Much more information on the harms and benefits of iron supplements should be provided to health care professionals, pharmacists and pregnant women, so they can make an informed decision on whether to take them.

## Caption Electronic Supplementary Material

Supplemental table 1: Percentage of anemia at the first and second blood measurement. Supplemental table 2: Costs during pregnancy for the three most popular products

Supplemental Table 2
