# Supplementary material for: How many women take oral supplementation in pregnancy in Austria? Who recommended it? A cross-sectional study
Source: Wien Klin Wochenschr. 2019 May 16;131(19):462–7. doi: 10.1007/s00508-019-1502-9 (PMC6795630; doi:10.1007/s00508-019-1502-9)
Supplement: Supplementary file 1 — Supplemental table 1: Percentage of anemia at the first and second blood measurement. Supplemental table 2: Costs during pregnancy for the three most popular products [file 508_2019_1502_MOESM1_ESM.docx]

| Characteristics (n=122) | n (%) |
| --- | --- |
| **Anemia** |  |
| Only detected in first blood test | 1 (0.8%) |
| Only detected in second blood test | 8 (6.6%) |
| At first and second blood tests | 1 (0.8%) |
| No anemia | 112 (91.9%) |
