# Supplementary material for: How many women take oral supplementation in pregnancy in Austria? Who recommended it? A cross-sectional study
Source: Wien Klin Wochenschr. 2019 May 16;131(19):462–7. doi: 10.1007/s00508-019-1502-9 (PMC6795630; doi:10.1007/s00508-019-1502-9)
Supplement: Supplementary file 2 — Supplemental Table 2 [file 508_2019_1502_MOESM2_ESM.docx]

| Brandname | Price per day | 1) Costs during pregnancy | 2) Costs during pregnancy |
| --- | --- | --- | --- |
| Femibion 1 (Merck, Darmstadt, Germany) | €0.63 - 0.7 | €158.76 – 176.4 | €110.25 - 122.5 |
| Femibion 2 (Merck, Darmstadt, Germany) | €0.55 – 0.61 | €138.6 – 153.72 | €96.25 - 106.75 |
| Elevit pronatal (Bayer, Leverkusen, Germany) | €0.61 | €163.72 | €106.75 |
| Pregnavit SelectPhase II (Ratiopharm, Ulm, Germany) | €0.49 – 0.54 | €123.48 – 155.52 | €85.75 - 94.5 |

1. Started taking medication during pregnancy and before first blood sample was taken (from the 5^th^ week of pregnancy)
2. Started taking medication during pregnancy but after first blood sample was taken (from the 16^th^ week of pregnancy)
Recommended retail prices in pharmacies in Austria, March 2017
